# Inferring biomarkers for *Mycobacterium avium* subsp. *paratuberculosis* infection and disease progression in cattle using experimental data

**DOI:** 10.1038/srep44765

**Published:** 2017-03-20

**Authors:** Gesham Magombedze, Tinevimbo Shiri, Shigetoshi Eda, Judy R. Stabel

**Affiliations:** 1 Center for Infectious Diseases Research and Experimental Therapeutics, Baylor Research Institute, Baylor University Medical Center, Dallas, TX, USA; 2 Department of Infectious Disease Epidemiology. Imperial College London, UK; 3 National Institute for Mathematical and Biological Synthesis, University of Tennessee, Volunteer Blvd, Suite 106, Knoxville, TN, 37996, USA; 4 Warwick Clinical Trials Unit, Warwick Medical School, University of Warwick, Coventry, UK; 5 Department of Forestry, Wildlife, and Fisheries, University of Tennessee, Knoxville, TN 37996-1527, USA; 6 USDA-ARS, National Animal Disease, Ames, IA 50010, USA

## Abstract

Available diagnostic assays for *Mycobacterium avium* subsp. *paratuberculosis* (MAP) have poor sensitivities and cannot detect early stages of infection, therefore, there is need to find new diagnostic markers for early infection detection and disease stages. We analyzed longitudinal IFN-γ, ELISA-antibody and fecal shedding experimental sensitivity scores for MAP infection detection and disease progression. We used both statistical methods and dynamic mathematical models to (i) evaluate the empirical assays (ii) infer and explain biological mechanisms that affect the time evolution of the biomarkers, and (iii) predict disease stages of 57 animals that were naturally infected with MAP. This analysis confirms that the fecal test is the best marker for disease progression and illustrates that Th1/Th2 (IFN-γ/ELISA antibodies) assays are important for infection detection, but cannot reliably predict persistent infections. Our results show that the theoretical simulated macrophage-based assay is a potential good diagnostic marker for MAP persistent infections and predictor of disease specific stages. We therefore recommend specifically designed experiments to test the use of a based assay in the diagnosis of MAP infections.

*Mycobacterium avium* subsp. *paratuberculosis* (MAP) is the causative agent of paratuberculosis (Johne’s disease [JD]), a chronic enteric wasting disease of ruminant animals with worldwide distribution^1^,^2^. As the disease progresses, there is loss in milk production, increased incidence of mastitis and infertility, which lead to early culling^3^. This causes a significant economic loss in beef and dairy farming^4^,^5^. The lack of a complete understanding of the host immune responses against this pathogen has hindered the development of effective control and diagnostic tools.

Transmission of JD can occur by ingestion of the bacterium through manure-contaminated feedstuffs and pastures or by colostrum and milk, passed from an infected dam to a calf 6,7. Upon ingestion, MAP bacilli target the small intestine where they are taken up by M cells and enterocytes, and subsequently engulfed by submucosal macrophages^8^,^9^,^10^,^11^. The immune response associated with MAP infection is complex and currently it is not completely understood. Previous studies have shown that, initially, animals control infection with a Th1 response predominated by the secretion of cytokines such as IFN-γ that activate macrophages to kill the intracellular bacteria^12^. As disease progresses and clinical manifestations begin to occur, there is a shift from a cell-mediated Th1 response to a non-protective Th2 response characterized by antibody titers to MAP. Some animals that demonstrate clinical signs of disease may have both Th1/IFN-γ and Th2-mediated immune responses (ELISA antibodies), whereas other clinical animals seem to lose Th1-mediated immunity^13^,^14^. This suggests that there are other mechanisms involved in T cell function during disease progression other than simply the shift to a Th2 response. How Th1 and Th2 responses characterize infection progression/disease, which is corroborated by bacteria shedding is still a riddle. In general it still remains to be clearly explained how MAP infection/disease progress differently in animals. There are several studies that give a detailed account on how the disease rapidly progresses in some of animals, while subclinical infection persists^15^,^16^,^17^.

Markers that can accurately define disease progression for MAP infection are still to be identified. Currently, the diagnosis of MAP is based mainly upon the detection of the bacterium in feces by culture or PCR and by ELISA detection of MAP-specific antibodies. Also, a cell mediated immune assay based on IFN-γ stimulation is used^18^,^19^. Detection of animals in the subclinical stage of infection can be difficult as these animals typically excrete MAP in low numbers and have not yet developed measurable antibody titers to MAP^20^,^21^,^22^. Research to find predictors and markers of disease progression or to identify antigens that can be used to accurately predict (or diagnose disease) is still lacking. For example, the IFN-γ assay, a measure of Th1-mediated immune response, is normally evident during the subclinical stage of the infection/disease^23^,^24^ and is considered a marker of early infection. In contrast, antibody detection assays such as the ELISA are used to assess Th2-mediated immune responses that are predominant in the late stages of infection and are more closely associated with mid- to advanced clinical disease. Assays of these types may be used to predict disease outcome or disease status before fecal shedding of MAP becomes evident.

Bacterial shedding is an important diagnostic parameter and a measure for disease status in *paratuberculosis*^18^,^25^,^26^. However, it does not provide insights into immune responses that are engaged. MAP shedding in the feces of infected animals is a primary route through which the environment can become contaminated. However, shedding and potentially transmission could be ongoing well before fecal culture tests yield positive results. Animals in the subclinical stage of infection shed few MAP organisms in their feces and do so intermittently throughout this phase of infection. In contrast, animals in advanced stages of infection (clinical) will shed MAP at high levels and shed on a continuous basis^14^,^25^,^27^. Therefore, other disease predictors are required, since fecal culture and PCR are more suitable for detection of advanced infections. Substantial evidence indicates that bacterial shedding into the feces is correlated with proliferation of MAP in the intestinal wall^17^. In light of this, it is important to realize that there are several factors that influence MAP bacterial shedding as reviewed in Koets *et al*.^17^ and inferred in modeling studies^28^,^29^,^30^. These may include the lifespan of macrophages in the host and the replication of bacteria within the host cells; the recruitment of monocytes to the site of infection, the integrity of the epithelial cell lining which may affect shedding of MAP to the lumen, and the level (weak, intermediate or robust) and kind of immune response expressed (humoral or cell mediated).

In this study, we used mathematical models to explain how Th1/IFN-γ and Th2 (ELISA antibodies) immune responses and fecal shedding can be used to predict MAP infection and stages of JD progression. Our model is framed on the current knowledge that the Th1 responses are protective against MAP infection, while the Th2 responses are not. Since bacterial shedding is typically used to categorize the stages of JD, we based our analyses on a dataset of IFN-γ and ELISA results from 57 cattle that showed different patterns of bacterial shedding. By grouping the animals according to the stage-specific shedding patterns of MAP disease, we sought to predict how Th1 and Th2 responses could explain these patterns and hence identify Th1 and Th2 profiles that can be used to predict the stage of infection and hence disease outcome. Finally, we then used the parameterized models to simulate alternative diagnostic assays based on infected macrophages since MAP primarily infect, persist, and replicate inside macrophages.

## Methods and Materials

### Experimental data

Holstein cows (n = 57) used in the present study were purchased off-site from dairy herds with known incidence of Johne’s disease or born to previously purchased infected dams and raised on-site at the National Animal Disease Center (Ames, IA). Cows had a median age of 3.00 years (interquartile range (IQR) of 3) at the initiation of the sampling period and median age of 5.92 years (IQR 3.98) at the termination of sampling. Years of collection ranged from 1 to 9 years, spanning the time period from 1998 to 2012, with median collection period per cow of 2.95 years (IQR 3.13) and samples were collected at 6 month intervals. Infection was characterized during the study period by three main diagnostic tools used for detection of JD in dairy herds. All animal related procedures were approved and performed in accordance with the guidelines and regulations of the IACUC (National Animal Disease Center, Ames, Iowa).

### Diagnostic testing

#### Fecal shedding

Infection was monitored bacteriologically for the fecal shedding of MAP using a modified centrifugation and a double-decontamination method as previously described by Stabel 1997^31^. Briefly, 2 g of fecal samples were decontaminated overnight at 39 °C in 0.9% hexadecylpyridinium chloride (HPC), followed by centrifugation at 1700 × g for 20 min the following day. Pellets were resuspended in 1 ml of an antibiotic solution containing 100 μg/ml naladixic acid (Sigma Chemical Co., St. Louis, MO), 100 μg/ml vancomycin (Sigma) and 50 μg/ml amphotericin B (Sigma). After overnight incubation at 39 °C, decontaminated samples (200 μl) were inoculated onto slants of Herrold’s Egg Yolk Medium (HEYM; BBLTM Herrold’s Egg Yolk Agar Slants with mycobactin J, amphotericin, nalidixic acid, and vancomycin; Becton Dickinson and Co., Sparks, MD) in replicates of 4 and incubated for 12 weeks at 39 °C. Tubes were examined and colony counts enumerated every 4 weeks during the 12-week period. At 12 weeks, a final read was taken and colony count averaged across all 4 slants for each cow.

#### Whole blood interferon-gamma assay

The whole blood IFN-γ assay was performed during the study period as previously described by Stabel and Whitlock^32^. Briefly, 1 ml aliquots of whole blood obtained in sodium heparin vacutainer tubes (Becton-Dickinson) were pipetted into wells of 24-well tissue culture plates (Becton-Dickinson). Blood samples were cultured alone (non-stimulated) or with 10 μg/ml of a whole cell sonicate preparation of MAP (strain 19698, MPS, National Animal Disease Center). The MPS (*M. paratuberculosis* sonicate) was prepared by sonication of 1 ml volumes of MAP (1 × 10^9^/ml) in PBS at 25 W for 30 min (3 cycles of 10 min each) on ice. Samples were incubated for 18 hr at 39 °C in 5% CO_2_, humidified atmosphere. Following incubation plates were centrifuged at 500 × g for 15 min and plasma was harvested from each well. Plasma samples were frozen at −20 °C until analyzed for IFN-γ by ELISA using the Bovigam kit (Life Technologies, Carlsbad, CA). A sample was determined to be positive if the absorbance of the stimulated sample (MPS antigen) was 0.100 absorbance units greater than the absorbance achieved for the non-stimulated control well for that animal. This classification of positive reaction was extrapolated to similar interpretations reported by researchers who have used the IFN-γ assay for detection of tuberculosis in cattle^33^,^34^,^35^.

#### MAP antibody detection

The assay used to measure MAP-specific antibodies in serum was performed using a commercial kit according to manufacturer’s instructions (IDEXX, Westbrook, ME). Briefly, samples were diluted in sample diluent containing *M. phlei* to remove cross-reacting antibodies and then dispensed into 96-well plates coated with MAP antigen, along with positive and negative controls that were provided. Samples were incubated for 45 min at room temperature, plates were washed 3 times and horseradish peroxidase-conjugate was added to each well. After 30 min incubation at room temperature, plates were washed again and TMB (3,3′,5,5′-Tetramethylbenzidine) substrate was added. Plates were incubated for another 10 min at room temperature and a stop solution was added to each well. Plates were read at *A*_450 nm_. A sample to positive result ratio (S/P) was calculated according to kit instructions upon subtraction of background noise (negative control absorbance) from samples and positive controls. If the S/P ratio was greater than 0.25 then samples were considered positive for MAP antibody in the serum. Note that the cut-off for a positive result was modified to 0.700 in 2009 by IDEXX and some of the serum samples collected from cows in this study were analyzed with this higher cut-off. Modifications were made to improve specificity and did not impact sensitivity of detection of positive cows. Therefore, results would be comparable to the prior cut-off values used in earlier test kits.

#### Infection groups

**Figure 1:**
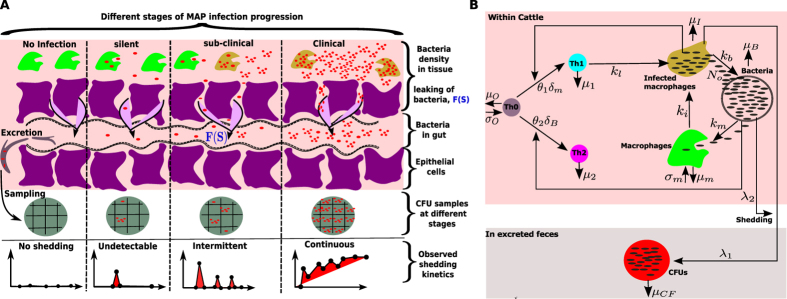
(**A**) Disease progression stages and the corresponding MAP shedding kinetics that can be detected in CFU sample’s at each stage. (**B**) Model diagram: Macrophages kill free bacteria at rate *k*_*m*_ and get infected at rate *k*_*i*_ giving rise to infected macrophages. Uninfected and infected macrophages have death rates given by *μ*_*m*_ and *μ*_*i*_, respectively. Infected macrophages burst at rate *k*_*b*_ and they release *N*_*o*_ bacteria at the same time. Th1 cells are assumed to kill infected macrophages at rate *k*_*l*_. IFN-γ and antibodies are assumed to be Th1 and Th2 subset surrogates, respectively. Both the population of infected macrophages and free bacteria are assumed to be the source of bacteria excreted in feces at rates λ_1_*F*_*m*_(*I*_*m*_) and λ_2_*F*_*B*_(*B*), respectively.

Cows were stratified into infection groups by monitoring fecal shedding of MAP by culture on HEYM as described above. For our criteria in this study, the level and consistency of MAP fecal shedding was used to categorize the cows into 4 different groups: 1) non shedding animals (shedding level-0, Group 0/no infection) included animals in which no shedding of MAP and immune stimulation was detected within the study period; 2) silent animals included cows that may have shed one time but at negligible levels (level-L, Group 1/silent); 3) asymptomatic animals (shedding level-M, Group 2/subclinical) included cows that shed intermittently and at low levels of MAP in their feces; 4) clinical animals included cows that consistently shed high levels of MAP in their feces for much of the study period (level-H, Group 3/clinical) (see Fig. 1A). We further defined the stratum as clinical cows were shedding more than 100 CFU per g of feces and presented with weight loss and intermittent diarrhea. Subclinically-infected cows were shedding less than 10 CFU/g of feces and were asymptomatic. The shedding levels 0, L, M, and H correspond to a continuum level of shedding <0.1, 0.1–0.35, 0.35–0.75, and >0.75, respectively after normalization (achieved through dividing all values by 100, therefore the value 1 corresponds to >=100 CFU/g and 0.1 represents 10 CFU/g).

### Theory of MAP shedding biology and how it correlates with immune responses

We made an assumption that infection begins when intestinal macrophages engulf MAP bacteria. Initially, macrophage seems to be able to control the infection, but at some point instead of suppressing intracellular MAP replication or killing the internalized bacteria, the bacteria will begin to replicate within the macrophages. This leads to the rupture of the highly infected macrophages, resulting in uptake of the expelled bacteria by new (nearby uninfected) macrophages. Then a cycle of macrophage infection by MAP commences. The host responds by mounting adaptive immune responses (cell-mediated (Th1) and humoral (Th2)) to try to control this infection process. In the model, IFN-γ is used as a surrogate for the Th1-type response, while the ELISA antibodies act as a surrogate for the Th2-type response.

Depending on the robustness of the stimulated immune response, the animals can clear the infection or prevent it (no infection, Fig. 1A). In this case no CFU will or can be detected in the feces. However, the observed transient antigen-specific IFN-γ responses may indicate that an infection event has occurred. The primed immune responses might not be strong enough to clear or prevent the infection from getting established, but may suppress it for an unpredictable duration of time. In this case, CFUs are not detectable by fecal testing, but immune response assays may indicate the presence of the infection (silent stage, Fig. 1A). Our theory is that if there is no infection in the host, antigen-specific immune responses should not be measurable. Any observable immune responses are an indicator of the presence of the infection in tissue, even when no bacterial shedding is detected. However, unlike in the silent stage, animals may slowly begin to present with sporadic positive fecal culture tests (subclinical stage, Fig. 1A). This stage is normally associated with high expression of Th1 immune responses compared to Th2 responses. Different studies have shown that^14^,^36^,^37^,^38^ the Th1 responses wane over time as Th2 responses begin to expand. Concurrent with the appearance of Th2 mediated immunity is a distinct continuous MAP shedding pattern that can be easily detected due to the high number of bacteria shed (clinical stage, Fig. 1A).

### Model development

We developed three models with different assumptions to explain the Th1/IFN-γ and Th2 (ELISA antibodies) immune response data, and the fecal shedding (CFU). In our models, we assumed that bacteria from both infected macrophages and free bacteria at the site of infection leaks into the gut via some mechanistic process. A certain fraction of infected macrophages transport the intracellular bacteria into the gut where some of them burst and release bacteria. Another fraction of free bacteria can migrate into the gut, see Fig. 1A. The general schematic presentation of mechanistic framework is shown in Fig. 1B with model equations given by system of equations (1, 2, 3, 4, 5, 6, 7), where we capture the dynamics of uninfected macrophages (*Mϕ*), infected macrophages (*I*_*m*_), free bacteria (*B*), naive T cells (*Th*_0_), Th1 (*Th*_1_) and Th2 (*Th*_2_) effector cells. The model assumes that the Th1 effector response kill infected macrophages (therefore protective), while Th2 response do not (therefore not protective). For simplicity, we assume that Th1 responses are stimulated by the population of infected macrophages while the Th2 response is stimulated by extra-cellular bacteria^29^,^30^. All the models assume similar cell biological interactions, however, they differ on how shedding can occur. Shedding can be explained and modeled by using (i) a dynamic model (system of ordinary differential equations (ODEs)), (ii) a stochastic dynamic model and (iii) a stochastic process based on a discrete logistic probability hazard function. All model parameters are defined in Table S1.


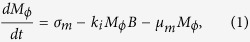



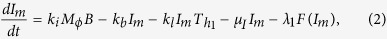



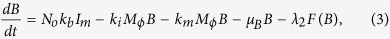



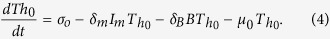



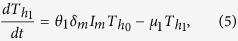



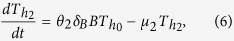



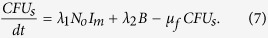


First, we model shedding as a continuous variable, i.e. the shedding functions 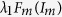 and 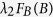 depend on state variables *I*_*m*_ and *B* with rate constants *λ*_1_ and *λ*_2_ (rates at which the bacteria either from infected macrophages or free bacteria leaks from the lamina propria into the gut, respectively). Secondly, to capture the fluctuations in the Th1, Th2 and CFU observations we added white noises to the corresponding equations, i.e., 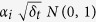, where *α*_*i*_ is the strength of the noise for each immune response, *δ*_*t*_ is the integration step size and *N*(0, 1), is a standard Gaussian random variable. Thirdly, we model shedding as a stochastic process, where the probability that an animal will shed depends on the level of expressed Th1 (IFN-γ), Th2 (ELISA) responses and other non-immune response related factors. For simplicity we assume a scaled logit model for the probability of shedding:


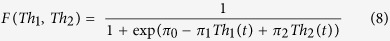


a joint probability density that an animal expressing IFN-γ (*Th*_1_) and ELISA (*Th*_2_) levels sheds. The intercept, *π*_0_ captures the bacteria shedding from any other factors not directly linked to the immune response variables. The slopes *π*_1_ and *π*_2_, represent the steepness of the shedding curve subject to Th1 and Th2 responses, respectively, which we assumed to be of different signs (*π*_1_ negative and *π*_2_ positive). This framework assumes that there is a direct link between *I*_*m*_ and *B* with CFU which can be explained by *Th*_1_ and *Th*_2_ concentrations (or relative concentrations). Also, shedding can be a result of other factors such as the assay error (noting that the CFU assay is 70% sensitive^39^,^40^,^41^) or other biological explanations. In the hybrid model, equation (7) is replaced by a discrete scaling


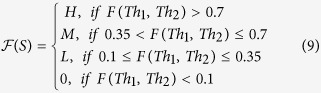


where H, M, L and 0 represent high, medium, low and no shedding, respectively.

### Model parameter estimation

We used the Markov Chain Monte Carle (MCMC) method based on a Bayesian framework implemented in the FME package in R^42^ to estimate model parameters (see Text S1 for more details on parameter selection for model fitting and the fitting procedure). We used a Gaussian likelihood to draw model parameter posteriors assuming uniform non-informative priors while the variances were regarded as nuisance parameters. The MCMC chain was generated with at least 100,000 runs when fitting data for each animal. Chain convergence was examined visually and with quantitative diagnostic tools in Coda R package (see Text S1 for more details). Uncertainty of each estimated parameter was evaluated by analyzing the MCMC chains by calculating the 2.5 and 97.5 quantiles of the chain around its median to give the 95% credible intervals (CrIs). The model baseline parameter values and priors are given in Table S1 and the estimated values in Table 1.

### Statistical analysis of associations between shedding and immune responses

We used three different statistical approaches to investigate the relationships between the cell-mediated immune response (IFN-γ/Th1) and the antibody immune response (ELISA/Th2) with MAP shedding (CFU). In the first approach, we calculated correlations between experimentally measured immune variables and the MAP CFUs. There is an understanding in this field that IFN-γ/Th1-type responses are protective while the antibody immune response/Th2 is not. We expected to see strong positive correlations between CFUs and the antibody immune response and a strong negative correlation between CFUs and IFN-γ. We evaluated the correlations before and after grouping the animals by MAP shedding stages as explained in Fig. 1A. We also analyzed correlations for animals that showed positive infection status, shown by either a positive IFN-γ assay or ELISA assay as well as the fecal culture assay. In the second method, we used generalized estimating equations (GEEs) to predict associations between immune response correlates and the CFU shedding. We evaluate the odds ratios and the adjusted odds ratios that an animal sheds bacteria in its feces given it had (i) a negative IFN-γ and ELISA status, (ii) a positive ELISA status only (iii) a positive IFN-γ status only, and (iv) a positive status for both the IFN-γ and ELISA assays. In this approach, the age at infection (or first sampling) was also analyzed to see if it had a significant contribution or influence on the shedding predictions for all animals and for those in specific shedding groups. Lastly, to predict causal relationships between the expressed immune responses and the cattle shedding dynamics at different stages of the disease/infection we used the dynamic models explained above. This method predicts mechanisms through which shedding occurs and how it changes with the expressed immune responses in a way that predicts shedding or disease outcome given a combination of expressed immune responses, which should explain (i) no infection, (ii) no shedding, (iii) intermittent shedding and (iv) continuous shedding.

### Evaluation of diagnostic assays using model simulations

We used the developed ODE model that predicts shedding mechanisms to simulate different assays and test if they can reliably reproduce the observed experimental assay results. A sample (or a value) is drawn from the model-simulated cell populations at different time intervals of infection: i) 0–6 months, ii) 6–12 months, iii) 12–18 months, and then (iv) 18–24 months. And then utilizes the average of the assay samples to generalize the evaluations. Assay cutoff values of 0.04 (for macrophages and Th1 cells), 0.1 (for Th2 cells), and 0.01 (for CFUs) are used to replicate the experimental assay cutoff to evaluate when the simulated assays are positive or negative. The simulated assays sensitivities are scored on a scale 0 to 1. A value >0.01 shows that the CFU based assay is positive, while the value >0.1 indicates that the Th2 assay is positive and values >0.04 predict Th1 and the theoretical macrophage based assays are positive (see Table S2 and Text S1 for details on the calculation of the sensitivity cut-off values). Also, we used the simulated assays to predict disease progression, hence, determine or diagnose the specific disease/infection stages. We set a cut off value of >0.1 to make assay comparisons since all cell populations were normalized.

## Results

### Disease class and stage classification

**Figure 2:**
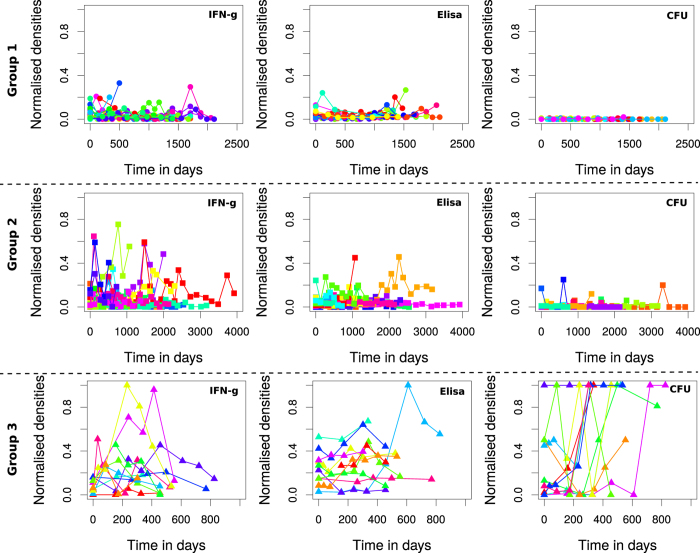
Classification of disease/infection groups. Time series kinetics of CFU shedding, IFN-γ and ELISA assay expressions for each of the 57 cattle grouped into separate categories based on immune and shedding patterns. Group 1 is classified as silent infections. In this group no shedding is observed and immune responses are not distinct and differently expressed. In Group 2 (sub-clinical infections), intermittent shedding is observed, while IFN-γ and ELISA test show differential expression of immune responses. Animals with high consistent CFU shedding and high IFN-γ and ELISA are categorized as Group 3, and are assumed to be in the clinical state.

Figure 2 shows different animals (n = 57) with MAP infection partitioned into 3 groups (no animal satisfied the criteria for Group 0). Group 1 is comprised of 25 animals (44%) that were negative for MAP fecal shedding. Within this group, both Th1 (IFN-γ) and Th2 (MAP antibodies) expressions are low (less than 0.2 and with an average of 0.1). No bacteria were detected from the fecal samples over time the animals were followed. In Group 2 (32%, 18/57), shedding is observed to occur at irregular intervals. However, a maximum level of 0.2 CFU shedding is observed, indicating that the animals are low intermittent shedders. The irregular low bouts of bacteria shedding are matched by different expression levels of Th1/Th2 responses. Some of the animals attain Th1 expression levels of 0.8, while the Th2 expression is relatively lower (with an average of about 0.2). Group 3 animals (24%, 14/57) are categorized by high and consistent bacterial shedding. Disparate CFU shedding patterns and different Th1/Th2 expressions are evident. In general, Th1 expression increases and peaks after about 200 days of assay (or age). It begins to decline after about 500 days. The expression pattern of the Th2 response does not change much, though some animals indicate an increasing trend with the duration of infection. The high CFU values are very distinct in this group and uniquely characterize animals that will rapidly develop clinical signs of JD.

### IFN-γ and ELISA assays are associated with MAP infection/disease progression

**Figure 3:**
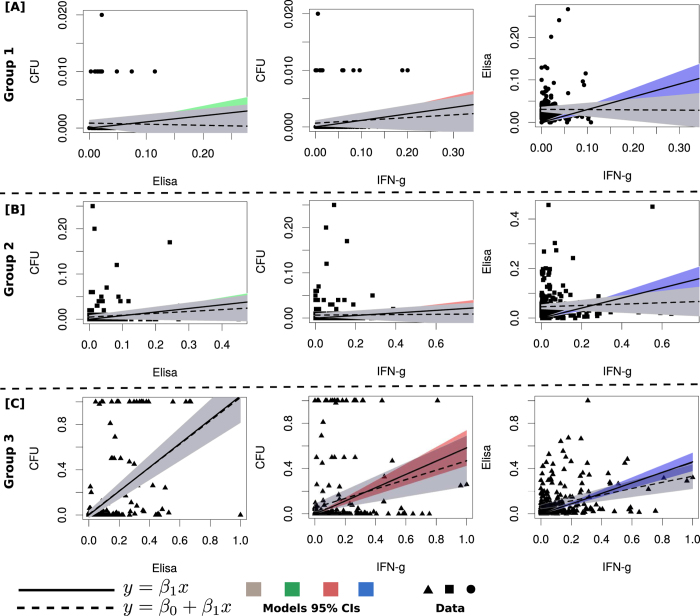
Immune markers as predictors of disease progression. Fitting linear models to determine the level of associations between the immune variables and the level of shedding for animals in the different groups. In Group 1 and Group 2 animals, weak linear relationships between CFU vs Th1, CFU vs Th2 and Th1 vs Th2, are predicted. In Groups 3, the ELISA assay is shown to be strongly correlated with MAP shedding, while IFN-γ is also positively correlated with the CFU and the ELISA assays. The data in each group are represented by a different shape (i) Group 1 a circle, (ii) Group 2, a square and Group 3, a triangle. The model fitted lines are represented by (i) a solid continuous lines (the model *y* = *β*_1_*x*) with different color shadings showing the 95% CIs and (ii) the broken lines (*y* = *β*_0_ + *β*_1_*x*) with the grey shading showing the 95% CIs.

In Fig. 3, Group 1 and Group 2 animals show similar lack of linear relationships between (i) CFUs and MAP antibodies, (ii) CFUs and IFN-γ and (iii) ELISA-antibodies and IFN-γ expression. In Group 1, the level of correlations (using the equation 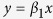, Table S3 or CFUs-vs-Th2, CFUs-vs-Th1 and Th1-vs-Th2 are *r* = 0.15 (p = 0.018), *r* = 0.21 (p = 0.0016), 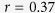 (p < 0.001), respectively. While in Group 2 the correlations are *r* = 0.22 (p < 0.001), *r* = 0.14 (p < 0.015), 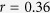 (p < 0.001), respectively. Model fits (Table S3) suggest no significant linear associations between the immune responses and bacteria shedding. The absence of bacteria shedding suggests the absence of infection (in Group 1) or an infection that cannot be confirmed with the current diagnostic tools. The low and intermittent shedding is accompanied by substantial Th1/Th2 immune correlates, which suggest a low or persistent MAP infection (Group 2 animals). Also, some animals in Group 2, express a relatively stronger Th1 response than a Th2 response. A high Th1 explains the absence of shedding and the intermittent shedding observed in some of the animals. However, fitting linear models to aggregated data smoothens the individual animal heterogeneities.

In Group 3, fecal shedding (CFU) has a relatively strong correlation with ELISA (Th2 immunity, *r* = 0.59 and p < 0.001) and also has a weak positive correlation with IFN-γ (Th1 response, *r* = 0.37 and p < 0.001), Table S3. Th1 and Th2 immune responses also have a positive association (*r* = 0.53, p < 0.001), contradicting the classical Th1/Th2 switch (which imply a negative association). The positive relationship shown in Fig. 2 (Group 3), is a summary description that fails to account for individual animal heterogeneities. But, also supports the notion that shedding can still occur even when a Th1-type response, which is believed to be protective, is expressed. Negative correlations between the Th1 and Th2 responses can be observed by looking at the regression slope when fitting was done using data for each individual animal, Figure S1. Similar results were also found when a generalized linear model was used to explore the association between shedding and the expressed immune responses. The computed odds ratios show a positive association between shedding and all immune responses (Table 2).

### Time evolution of Th1 and Th2 responses explain the kinetics of MAP infection and fecal shedding

**Figure 4:**
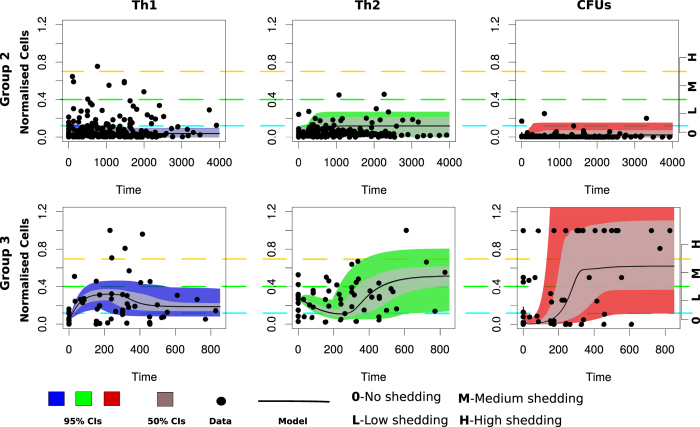
Comparing the dynamic model to Group 2 and Group 3 data. Model fitting to data using the ordinary differential equations dynamic model. The upper row shows Group 2 animals fitting while the lower row shows animals in Group 3. The solid lines represent the model fitting to the data (represented by the solid circles). The blue, green and red shadings represent the 95% CrIs while the grey shading represent the 50% Crls. Model parameters that were varied to generate the fits are given in Table 1 and the parameters that were fixed during model fitting are given in Table S1.

**Figure 5:**
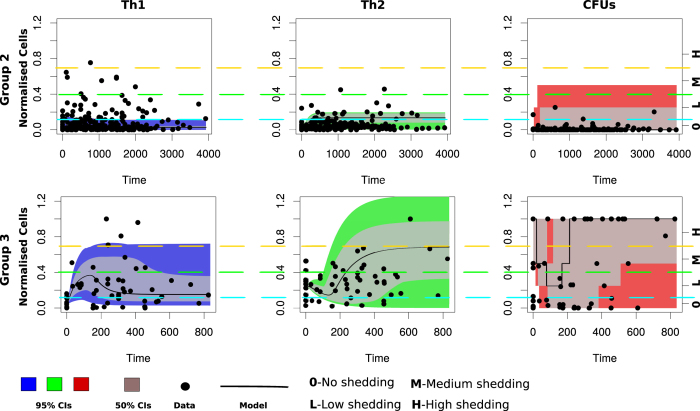
Comparing Group 2 and 3 animal data to the hybrid model. The Figure shows data fitting for Group 2 (upper row) and Group 3 (lower row) animals using a hybrid model. The solid lines represent the model fitting to the data (represented by the solid circles). CFU shedding is shown by a piecewise continuous logistic function. The color shadings (blue, green and red) represent the 95% CrIs and the 50% CrIs are shown by the grey shading. See Table 1 for the parameters that were varied during model fitting. Table S1 gives parameters that were kept invariant during model fitting.

Figures 4 and 5 show the time evolution of cell variables (Th1, Th2 and CFUs) using an ODE model and a hybrid model, respectively. In Fig. 4, CFU shedding was explained using a continuous process based on the ODE model. In Fig. 5, CFU shedding kinetics are explained using a piecewise continuous discrete logistic hazard function. However, in both figures and models the expressed Th1/Th2 responses are described with continuous time evolving variables using ODEs. Data fitting to Group 2 animals (Fig. 4, upper row) shows that low CFU shedding can be explained by low Th1 and Th2 responses. Fitting Group 3 animals (Fig. 4, lower row) predicts a high Th1 expression in the early stages of the infection (within the first 400 days), which is accompanied by a relatively low Th2 response. This pattern is reversed with disease progression. These Th1/Th2 dynamics are matched by CFU shedding that is initially low in the first 400 days, but subsequently increase as the Th1 response wanes. The observed MAP infection kinetics can be reproduced by fitting the parameters *k*_*i*_ (rate of macrophages infection by MAP), *k*_*b*_ (bursting of infected macrophages), *θ*_1_ (Th1 cell expansion), *θ*_2_ (Th2 cell expansion), *λ*_1_ (transportation of infected macrophages into the gut) and *μf* (life span of excreted bacteria), Table 1. In Group 3, high rates of macrophage infection, bursting, cell expansion, and shedding is predicted. While in Group 2, relatively low rates are predicted, Table 1. The magnitudes of the predicted rates explain the level (H-high, M-medium, and L-low or 0-the absence) of the expressed immune cells and bacteria in the fecal samples.

Figure 5, shows how the hybrid model fit Group 2 and Group 3 data. Similar to Fig. 4 (ODE model fitting), there was no difference in the infection and immune response parameters that were estimated. Fig. 5 clearly shows that when both Th1 and Th2 expressions are low, high CFU shedding is observed. We predict high CFU shedding in the early stages (within the first 200 days) of the infection in contrast to what we observe in Fig. 4. The ODE model fails to capture adequately all the bacteria data points at the early stages of the infection, Fig. 4. However, the hybrid model closely explains the shedding dynamics at all stages of the disease. Figure 5 shows that a high Th1 explains low shedding, while a high Th2 drives high shedding, and a balanced Th1/Th2 response is a predictor of low- to- medium shedding. In Group 2 animals, shedding varies from 0 (no)- to M (medium), Fig. 5. This is explained by a low and balanced combined Th1/Th2 response. However, in Group 3, we observe that the lack of Th1 immunity and a low Th2 immunity is a predictor of high shedding at the early stages of the infection. Bacteria shedding plummet with increasing Th1 expression, though this trend is reversed, as Th1/Th2 dominance is reversed (Figures S3, 4 and 5, Group 3). In Group 2 animals, Th1/Th2 responses remain lowly expressed, and does not change over time, hence predicting non-progressing persistent infections.

### Inference of disease stage specific markers and infection detection markers

**Figure 6:**
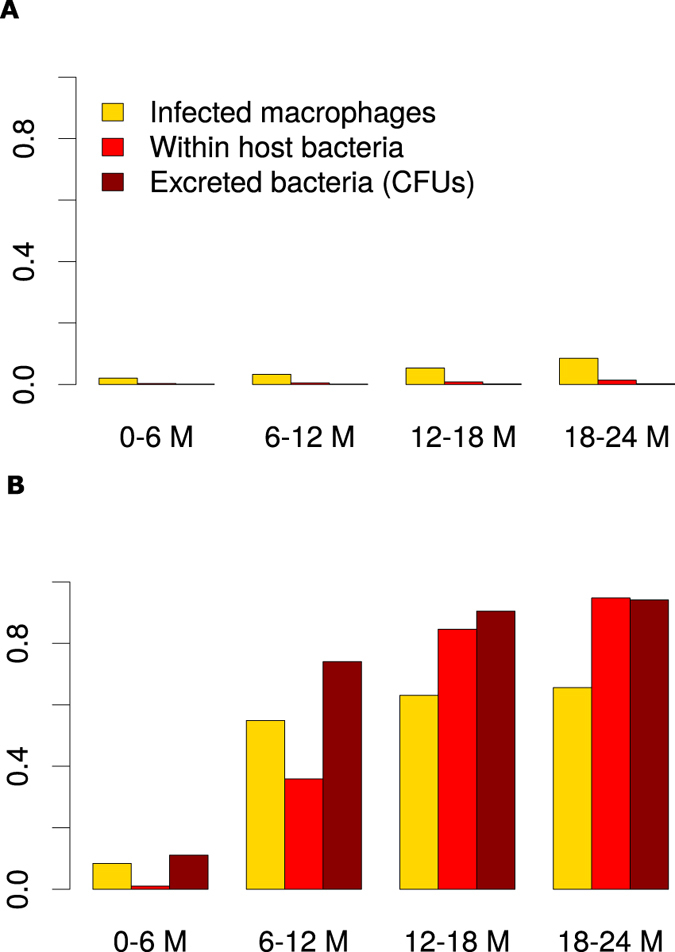
Assay simulation and comparison. Panel (A) shows that the population of infected macrophages is higher than the bacteria population. And these potentially stimulate the Th1 and Th2 responses noticed in Group 2 animals and their sensitivity increase over time. Panel (B) shows that in Group 3, there are more infected macrophages compared to within host extracellular bacteria in the first 12 months of infection, but later there are more free bacteria than infected macrophages as infection progresses. This figure was generated using the ODE model and the estimated parameters obtained through model fitting to data shown in Fig. 4.

**Figure 7:**
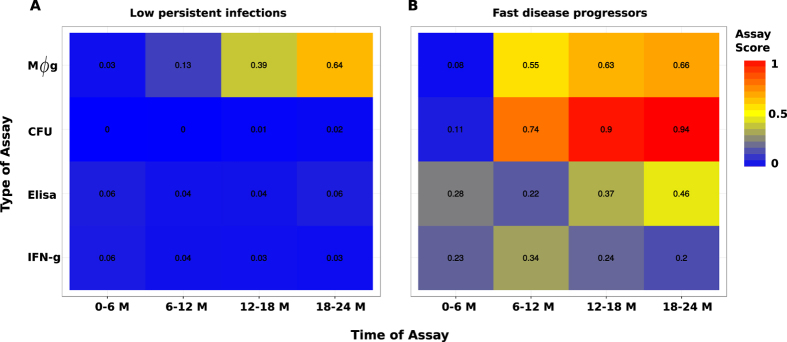
Theoretical assay evaluation. Panels (A) and (B) show results of the theoretical assay simulated using the basic model. Panel (A) shows assay evolution for non-progression infection, while (B) shows how the assays predict fast progression infections. Assay values were simulated using a six month moving average. The model estimated parameters were used to evaluate the sensitivity score of the different assays for each group.

Figure 6A shows that in non-progressing infections, the population of infected macrophages is higher than the population of within host and excreted bacteria. The population of infected macrophages slightly increases over time, but excreted bacteria remains undetectable, Figs 6A and 7A. Therefore, the invariant low Th1 and Th2 expressions predict infection persistence when no MAP excretion is evident. In Fig. 6B, we observe high levels of infected macrophages compared to the population of within host bacteria in the 1^st^ and 2^nd^ 6 month sliding windows. However, the level of excreted bacteria is always higher than that of infected macrophages. High populations of infected macrophages in the first 18 months of the infection correctly explains the infection biology of JD. MAP bacteria prefer to reside and replicate inside macrophages. This is the reason we see substantial infected macrophages when no bacteria is detectable (Fig. 6A). This result suggests that the presence of infected macrophages is a better predictor of slow progressing persistent infections, Fig. 7.

Figures 6B and 7A show that fecal shedding of MAP is the best predictor of disease progression followed by the theoretical macrophage-based assay, while the IFN-γ (Th1) assay is the least predictive within the first 12 months. Figure S2 shows a comparison of within host bacteria and the excreted bacteria, and the corresponding expressed Th1 and Th2 responses in the course of the infection. Figure 6 shows the corresponding population of infected macrophages and how they compare to the population of within host and excreted bacteria. Figure 7 shows the assay sensitivity scores in slow and fast disease progressors. We use 6-month sliding windows to illustrate how cell populations (averaged over the interval) evolve over time. Figures S2 and 6A show minimal Th1 and Th2 responses and undetectable levels of both within the host and excreted bacteria in Group 2 animals regardless of time. Figure 7A shows that IFN-γ, ELISA and CFU assays remain unchanged, therefore cannot be used to predict disease states. The IFN-γ and ELISA assays can only confirm the infection, but the CFU will falsely detect the absence of MAP infection. On the contrary, Fig. 6B shows infected macrophages, and within host and excreted bacteria, hence high sensitivity scores for all the assays are demonstrated (Fig. 7B). In the first 6 months, no within host bacteria are visible, but low CFU shedding is demonstrated. Th1 immunity wanes in the 3^rd^ (12–18 M) and 4^th^ periods (18–24 M), and this is accompanied by expansion of the Th2 response, as well as increases of within host and excreted bacteria.

### Comparison of infected macrophages based assay and IFN-γ, ELISA and fecal culture assays

Figure 7A shows that CFU, ELISA and IFN-γ assays have low sensitivity scores and are negative when a cut-off of 0.1 is set. This result agrees with the current knowledge of these assays as far as the diagnosis of JD is concerned, thereby explaining and confirming how unlikely it is to correctly detect silent and subclinical infections using the current diagnostic tools. However, it is interesting to note that an assay based on macrophages can predict MAP persistent infections (Fig. 7A) and has good sensitivity scores. Figure 7B shows that the current assays and the proposed macrophage based assay can reliably detect MAP infection. This prediction indicates that in fast progressing infections (Group 3 animals), the ELISA and IFN-γ assays can detect MAP infection within the first 6 months. The sensitivity of the IFN-γ assay increases within the first 12 months but decreases thereafter. The ELISA assay sensitivity is lowest between 6–12 months. Figure 7B shows that the fecal culture assay is the best predictor of disease progression, followed by the macrophage based assay. The ELISA and IFN-γ assays can also predict disease, but their sensitivities only change marginally, hence making them poor markers of disease progression, however, may be more useful in detecting MAP infection in its early stages than the fecal shedding assay. The increasing ELISA sensitivity scores make it a better predictor of JD progression than the IFN-γ assay, Fig. 7B.

## Discussion

Early diagnosis of MAP infections and the prediction of disease specific stages are of prime importance in the control of JD. Currently, the fecal culture assay is the gold standard for detection and confirmation of shedding animals and for characterizing disease states (silent, subclinical, clinical and advanced). The ELISA assay is an alternative diagnostic tool based on measures of MAP-specific antibodies. Both methods often misdiagnose animals in the early stages of the disease. The IFN-γ assay is also used to detect MAP infection and is based on measurement of antigen-specific release of IFN-γ in a whole blood assay. Based on these assays, MAP infection is classically explained by an early Th1-type immune response (IFN-γ) with a switch to a predominantly Th2 type (antibody/humoral response) accompanied by high bacteria shedding. In this study, we used mathematical models to explain and predict JD progression and disease-specific stages using the Th1 (IFN-γ) and Th2 (ELISA-antibodies) immune assays together with fecal culture. Further, we used an alternative theoretical simulated assay based on infected macrophages to predict disease and disease stages. As intracellular pathogens, mycobacteria like MAP, *Mycobacterium tuberculosis* (MTB), and *Mycobacterium avium* complex (MAC) preferentially reside and replicate inside macrophages. Bacteria can persist in the intracellular environment for protracted periods of time before shedding becomes evident. Based on our model simulation results we speculate that the theoretical simulated macrophage-based diagnostic assay can be a better predictor of MAP infection for animals with slow or persistent infections and also as a marker for JD stages in animals that progress to advanced disease rapidly.

Using generalized estimating equations, we predicted that (i) animals with high ELISA-antibody titers have the highest odds ratio of shedding MAP, (ii) animals with an IFN-γ positive status have the least odds ratio, and (iii) animals with both ELISA and IFN-γ positive statuses are intermediate shedders. The predicted odds ratios (Table 2) are corroborated by correlations between CFUs (fecal shedding) and the IFN-γ and ELISA assays, Fig. 2. These results suggest that substantial bacterial shedding still occurs even when a protective Th1 response is present. However, Th1 protection (negative Th1-CFU correlation) is observed in some of the animals, Figure S1. Analyzing data of animals in the group with slow and non-progressing infections revealed no significant linear relationships between the measured parameters. Also, aggregating data failed to account for heterogeneities between individual animals, but helped to summarize predictions for animals with similar shedding and immune response patterns.

Correlations and associations have limitations in making robust predictions since they cannot explain the time evolution of the infection. Therefore, we applied dynamic mechanistic models to describe how the immune system interacts with the MAP pathogen. Our models correctly explain and mimic patterns observed in the data. Our model fitting to data predicts, (i) infection mechanisms represented by the rate of macrophage infection by MAP bacteria (*k*_*i*_), the bursting rate (*k*_*b*_), (ii) immune response mechanisms (clonal expansion after Th1 and Th2 lineage commitment), and (iii) the trafficking of infected macrophages into the lumen (*λ*_1_) and the life span of excreted bacteria (*μf*) as the main mechanisms that are necessary to explain the data. Variation in the magnitude of the estimated parameters explained the disparate immune responses and MAP bacteria shedding patterns between animals in Groups 2 and 3. Animals in Group 3 are explained by high macrophage infection rates (*k*_*i*_), stronger Th1 and Th2 expansion, high trafficking of infected macrophages into the gut and slow decay rate of excreted bacteria. The low cell populations in Group 2 animals are explained by rapid bacteria decay rates, low Th1 and Th2 expansion rates and slow rates of macrophage infection. We predict relatively equal burst rates for infected macrophages between Group 2 and Group 3 animals, Table 1. We predict stronger Th2 expansion than the Th1 expansion in both groups. High infection of macrophages will initially lead to the increase of infected macrophages, hence selecting for Th1 differentiation. However, as the population of infected macrophages increases free bacteria will eventually accumulate as the population of uninfected macrophages gets depleted. This in turn skews the Th1/Th2 differentiation pathway along the Th2 lineage, hence the loss of the initially-expressed Th1 response. However, the slow persistent infections are characterized by consistent low expressions of Th1/Th2 responses and undetectable levels of bacteria. The lower infection rate of macrophages explains the low population of infected macrophage hence a weak stimulation of the Th1 response. The substantial populations of uninfected macrophages that can kill MAP prevent the accumulation of extracellular bacteria, leading to a weak induction of the Th2 response. A low population of infected macrophages and their slow leakage into the gut is a plausible explanation for low and undetectable shedding observed in persistent infections.

**Figure 8:**
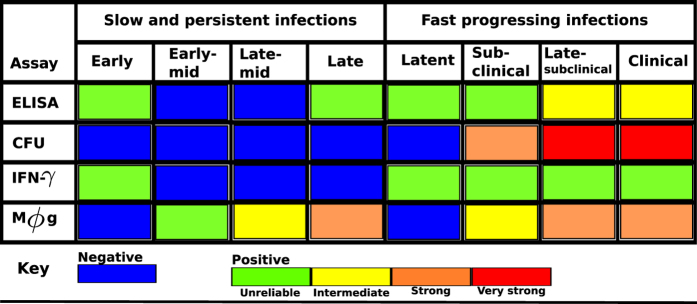
Assay summary diagnostic interpretation. The ability of each separate assay to detect MAP infection and JD progression stages in non-progressing infections and fast progressing infections are shown. The blue color represents a negative assay result, the green color represents an unreliable assay result, and the colors, yellow, brown, and red, stand for intermediate, strong and very strong assay prediction results, respectively.

Macrophages are important cells in MAP infection and for several other mycobacterial pathogens such as MTB and MAC. The pathogen prefers to reside and replicate intracellularly and have the ability to evade and subvert immune surveillance and signaling pathways to avoid being killed^43^,^44^,^45^,^46^. MAP and MTB were shown to prevent phagosome-lysosome fusion, enabling it to adapt and persist in macrophage vacuoles^43^,^44^,^45^,^46^,^47^. The pathogens are anti-apoptotic, therefore keeping the host cell alive to avoid anti-microbial effects of apoptosis and pro-necrotic killing of the host macrophage to allow infection of neighboring cells. Our model results corroborate this understanding (Fig. 6A). We see a substantial number of infected macrophages and undetectable levels of within host and excreted bacteria. The continual presence of a few infected macrophages explains MAP persistence. In non-progressing infections, there is no evidence of shedding, hence the failure of fecal assays to detect the infection. These results, Figs 7 and 8, render the use of macrophage-based assays in the diagnosis of MAP infections attractive, demonstrating that the simulated macrophage assay had better disease prediction sensitivity scores than the Th1 and Th2 assays.

Recently, there have been some interesting findings in the search of alternative biomarkers for diagnosing mycobacterial infections. The studies^48^,^49^,^50^ showed that there are several mycobacterial products, mostly proteins and peptides that are secreted by MTB and MAC infected macrophages that can be detected in the exosomes from host serum. The secreted exosomes contain glycopeptidolipids that can be transferred from infected to uninfected macrophages hence stimulating a pro-inflammatory response in resting macrophages. The proteins associated with the exosomes or the mycobacterial products are known to contribute to the intracellular survival of MTB^51^,^52^,^53^. Additionally, studies^54^ have proposed exosomes as potential biomarkers across a spectrum of TB disease states (latent and active states), which are synonymous with persistent (latent/silent or slow progressing) and rapidly progressing infections in JD. When we evaluated the sensitivity of our simulated macrophage assay in the prediction of different JD states (Fig. 7), we found that it was a good predictor of a persistent infection, suggesting it would be more useful for detection of latent/silent infections than the current diagnostic assays. However, this result requires specifically designed experiments for validation. We also illustrated that the simulated macrophage assay is a better predictor for JD stages in rapidly progressing infections than the Th1/Th2 immune assays. Fernandez *et al*.^55^ recently reported that macrophages in focal lesions have few intracellular MAP polarized toward a M1 phenotype, while the cells in multibacillary lesions showed M2 (anti-inflammatory) phenotype, supporting our prediction that a macrophage assay can be used for evaluation of JD status. However, this will require sampling of macrophages from the site of infection, which is not practical. Since JD-status(resistance)-dependent difference of macrophage phenotype (cytokine expression and apoptosis) was reported in macrophages derived from circulating monocytes, it would be of interest to examine if such ante-mortem macrophage assay has a value in diagnosis of JD status^56^ or test for exosomes in serum that are secreted by infected macrophages.

To avoid the limitations associated with a specific type of model choice in making predictions we used a suite of dynamics models and standard statistical approaches. The dynamic models explain the data well and the association analysis corroborates these results. Of all the models used, the hybrid model accounts for the CFU shedding kinetics at the different disease stages more elegantly. And adding stochastic noise to the ODE model reasonably explains the erratic fluctuations observed in the measurements. Our model results illustrate that (i) a high or low Th1 expression with a low Th2 expression accompanied by no or by low CFU shedding predicts silent/latent disease, (ii) a balanced Th1/Th2 response with substantial fecal shedding is a predictor for subclinical (and early clinical) disease. (iii) The clinical stage of the disease is predicted by a high Th2 expression, high shedding and a less affirmative Th1 assay. Our results indicate that the Th1 immune assay is not a good marker for disease progression, Th1/Th2 and fecal assays are not reliable in predicting slow or non-progressing persistent infections, and that Th1 and Th2 assays have low sensitivities and remain invariant over time, hence making prediction of persistent infections highly unlikely. In retrospect, the invariant evolution of these markers can be used as a cue for predicting**/**detecting persistent infections (non-progressing infections), while increasing ELISA, macrophage and CFU sensitivity scores detect progressing infections. Hypothetically, these assays can be used to detect non-infected animals. When, the IFN-γ and the macrophage based assays remain negative after the first 6 months of testing, it could be interpreted as the diagnosis of no infection.

In summary, we present findings that suggest that a macrophage based assay is a good diagnostic assay for persistent infections and an alternative predictor for JD specific stages in fast progressing infections. We, also show that the current Th1 and Th2 immune markers are important in MAP diagnosis, but are unreliable in predicting persistent infections. Our results also confirm that the fecal test is the best marker for MAP infection detection and for predicting JD stages in rapidly progressing infections, however, in slow or non-progressing infections it is of no use and the macrophage assay could be a better marker.

## Additional Information

**How to cite this article:** Magombedze, G. *et al*. Inferring biomarkers for *Mycobacterium avium* subsp. *paratuberculosis* infection and disease progression in cattle using experimental data. *Sci. Rep.* **7**, 44765; doi: 10.1038/srep44765 (2017).

**Publisher's note:** Springer Nature remains neutral with regard to jurisdictional claims in published maps and institutional affiliations.

## Supplementary Material

Supplementary Information

## Figures and Tables

**Table 1:** Estimated parameters.

Parameter	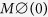	*I*_*m*_(0)	B(0)	*k*_*i*_	*k*_*b*_	*θ*_1_	*θ*_2_	*λ*_1_	*μ*_*f*_	
ODE Model										
Group 2	478.5(48.3, 950.7)	9.86(1.1, 19.1)	1.3(0.2, 2.7)10^-5^	3.8(0.4, 6.2)10^-4^	4.5(0.4, 7.4)10^-4^	45.58(4.7, 101.1)	128.29(13.0, 229.4)	1.4(0.15, 2.9)10^-4^	0.3886(0.03, 0.938)	
Group 3	39.18(34.2, 46.8)	1.41(1.1, 1.6)	1.19(0.85, 1.52)	1.3(0.4, 3.5)10^-3^	5.5(4.5, 7.4)10^-4^	386.24(217.8, 534.4)	607.2(390.0, 769.2)	1.4(0.26, 2.5)10^-3^	0.1493(0.0275, 0.2142)	
Hybrid Model								*π*_0_	*π*_1_	*π*_2_
Group 2	48.66(17.9, 75.7)	0.98(0.17, 1.80)	0.98(0.12, 1.74)	2.4(0.5, 3.7)10^-3^	0.9(0.1, 13.8)10^-4^	102.54(20.3, 174.4)	103.88(10.7, 182.0)	0.0105(0.0018, 0.02)	0.2279(0.057, 0.431)	0.01(0.002, 0.02)
Group 3	51.56(11.4, 93.4)	1.0(0.17, 1.80)	0.3(0.07, 0.5)	2.1(0.5, 3.6)10^-3^	0.8(1.7, 13.3)10^-4^	472.15(114.7, 9169)	584.32(106.4, 905.3)	0.0114(0.0024, 0.02)	0.0859(0.018, 0.18)	0.11(0.027, 0.18)

Estimated parameters through fitting the models to the grouped animal data. For each estimated parameter a 95% credible interval (CrIs) is given.

**Table 2:** Association of shedding and positive immune responses in 57 animals followed between 1 to 9 years (with a median follow up time of 2.95 years).

Characteristic		Shedding status in each group (CFU)		
		All animals	Group 2	Group 3
Age at baseline	OR (95% CI)	1.17 (1.01–1.37)	1.07 (0.78–1.47)	1.07 (0.83, 1.37)
	AOR (95% CI)	1.08 (0.94–1.24)	1.08 (0.86–1.36)	0.99 (0.80–1.23)
Interferon gamma (IFN-*γ*) status	OR (95% CI)	2.13 (1.45–3.13)	1.67 (0.95–2.91)	2.96 (1.22–7.19)
	AOR (95% CI)	1.75 (1.02–2.99)	1.42 (0.72–2.77)	0.54 (0.09–3.44)
Elisa status	OR (95% CI)	6.81 (3.87–12.0)	3.78 (1.40–10.2)	3.05 (1.02–9.15)
	AOR (95% CI)	3.97 (1.53–10.3)	2.65 (9, 55–12.9)	0.66 (0.13–3.31)
ELISA and INF-*γ* status	OR (95% CI)	9.09 (5.18–16.0)	5.78 (2.24–14.9)	3.78 (1.44–9.90)
	AOR (95% CI)	2.30 (0.76–6.94)	2.68 (0.41–17.6)	7.07 (0.81–61.7)

The estimated odds ratios (effects) are based on generalized estimating equations. INF-*γ* status was defined as a categorical variable with changes greater than 0.10 deemed positive and those less than 0.10 negative. ELISA status was defined as a categorical variable with antibody changes greater than 0.25 deemed positive and those less than 0.25 negative. CFU was categorized as shedding (values greater or equal to 1) and no shedding (values equal to zero). Group 2: animals with a combined immune response. Group 3: animals with progression of infection. OR: adds ratio; AOR – adjusted odds ratio.

## References

[b1] Stabel J. R. Johne’s disease: a hidden threat. Journal of dairy science 81, 283–288 (1998). 9493105 10.3168/jds.S0022-0302(98)75577-8

[b2] Sweeney R. W., Collins M. T., Koets A. P., McGuirk S. M. & Roussel A. J. Paratuberculosis (Johne’s Disease) in Cattle and Other Susceptible Species. J Vet Intern Med 26, 1239–1250 (2012). 23106497 10.1111/j.1939-1676.2012.01019.x

[b3] Lombard J. E. . Herd-level prevalence of Mycobacterium avium subsp. paratuberculosis infection in United States dairy herds in 2007. Prev Vet Med 108, 234–238 (2013). 22979969 10.1016/j.prevetmed.2012.08.006

[b4] Ott S. L., Wells S. J. & Wagner B. A. Herd-level economic losses associated with Johne’s disease on US dairy operations. Prev Vet Med 40, 179–192 (1999). 10423773 10.1016/s0167-5877(99)00037-9

[b5] Brett E. Johne’s disease: an economic evaluation of control options for the New Zealand livestock industries . Agriculture New Zealand, Feilding, New Zealand (1998).

[b6] Stabel J. R., Bradner L., Robbe-Austerman S. & Beitz D. C. Clinical disease and stage of lactation influence shedding of Mycobacterium avium subspecies paratuberculosis into milk and colostrum of naturally infected dairy cows. Journal of dairy science 97, 6296–6304 (2014). 25064655 10.3168/jds.2014-8204

[b7] Stabel J. R. Pasteurization of colostrum reduces the incidence of paratuberculosis in neonatal dairy calves. Journal of dairy science 91, 3600–3606 (2008). 18765618 10.3168/jds.2008-1107

[b8] Momotani E., Whipple D. L., Thiermann A. B. & Cheville N. F. Role of M cells and macrophages in the entrance of Mycobacterium paratuberculosis into domes of ileal Peyer’s patches in calves. Vet Pathol 25, 131–137 (1988). 3363791 10.1177/030098588802500205

[b9] Ponnusamy D., Periasamy S., Tripathi B. N. & Pal A. Mycobacterium avium subsp. paratuberculosis invades through M cells and enterocytes across ileal and jejunal mucosa of lambs. Res Vet Sci, S0034–5288 (2012). 10.1016/j.rvsc.2012.09.023 23122809

[b10] Sigurethardottir O. G., Valheim M. & Press C. M. Establishment of Mycobacterium avium subsp. paratuberculosis infection in the intestine of ruminants. Adv Drug Deliv Rev 56, 819–834 (2004). 15063592 10.1016/j.addr.2003.10.032

[b11] Sigur-Dardottir O. G., Press C. M. & Evensen O. Uptake of Mycobacterium avium subsp. paratuberculosis through the distal small intestinal mucosa in goats: an ultrastructural study. Vet Pathol 38, 184–189 (2001). 11280374 10.1354/vp.38-2-184

[b12] Stabel J. R. Transitions in immune responses to Mycobacterium paratuberculosis. Vet Microbiol 77, 465–473 (2000). 11118731 10.1016/s0378-1135(00)00331-x

[b13] Stabel J. R., Bannantine J. P. & Hostetter J. Mycobacterium avium subsp. paratuberculosis infection, immunology and pathology of livestock. Centre for Agriculture and Biosciences International 512–537 (2015).

[b14] Begg D. J. . Does a Th1 over Th2 dominancy really exist in the early stages of Mycobacterium avium subspecies paratuberculosis infections? Immunobiology 216, 840–846 (2011). 21281979 10.1016/j.imbio.2010.12.004

[b15] Mitchell R. M. . Differences in intermittent and continuous fecal shedding patterns between natural and experimental Mycobacterium avium subspecies paratuberculosis infections in cattle. Veterinary Research 46, 66 (2015). 26092571 10.1186/s13567-015-0188-x PMC4474556

[b16] Schukken Y. H. . Longitudinal data collection of Mycobacterium avium subspecies Paratuberculosis infections in dairy herds: the value of precise field data. Veterinary Research 46, 65 (2015). 26092492 10.1186/s13567-015-0187-y PMC4474331

[b17] Koets A. P., Eda S. & Sreevatsan S. The within host dynamics of Mycobacterium avium ssp. paratuberculosis infection in cattle: where time and place matter. Veterinary research 46, 1–17 (2015). 26092382 10.1186/s13567-015-0185-0 PMC4473847

[b18] Collins M. T., Gardner I. A., Garry F. B., Roussel A. J. & Wells S. J. Consensus recommendations on diagnostic testing for the detection of paratuberculosis in cattle in the United States. J Am Vet Med Assoc 229, 1912–1919 (2006). 17173528 10.2460/javma.229.12.1912

[b19] Wadhwa A., Hickling G. J. & Eda S. Opportunities for improved serodiagnosis of human tuberculosis, bovine tuberculosis, and paratuberculosis. Veterinary medicine international 2012 (2012). 10.1155/2012/674238 PMC3375143 22720192

[b20] Facciuolo A., Kelton D. F. & Mutharia L. M. Novel secreted antigens of Mycobacterium paratuberculosis as serodiagnostic biomarkers for Johne’s disease in cattle. Clinical and Vaccine Immunology 20, 1783–1791 (2013). 24089453 10.1128/CVI.00380-13 PMC3889510

[b21] Scott M. C. . Absorbed EVELISA: a diagnostic test with improved specificity for Johne’s disease in cattle. Foodborne Pathogens and Disease 7, 1291–1296 (2010). 20704508 10.1089/fpd.2010.0541

[b22] Leite F. L., Reinhardt T. A., Bannantine J. P. & Stabel J. R. Envelope protein complexes of Mycobacterium avium subsp. paratuberculosis and their antigenicity. Veterinary microbiology 175, 275–285 (2015). 25500374 10.1016/j.vetmic.2014.11.009

[b23] Stabel J. Host responses to Mycobacterium avium subsp. paratuberculosis: a complex arsenal. Animal health research reviews 7, 61–70 (2006). 17389054 10.1017/S1466252307001168

[b24] Mortier R. A. . Dose-dependent interferon-gamma release in dairy calves experimentally infected with Mycobacterium avium subspecies paratuberculosis. Veterinary immunology and immunopathology 161, 205–210 (2014). 25190508 10.1016/j.vetimm.2014.08.007

[b25] Whitlock R. H. & Buergelt C. Preclinical and clinical manifestations of paratuberculosis (including pathology). Veterinary Clinics of North America-Food Animal Practice 12, 345–356 (1996). 10.1016/s0749-0720(15)30410-2 8828109

[b26] Gardner I. A. . Consensus-based reporting standards for diagnostic test accuracy studies for paratuberculosis in ruminants. Preventive veterinary medicine 101, 18–34 (2011). 21601933 10.1016/j.prevetmed.2011.04.002

[b27] Kurade N. P., Tripathi B. N., Rajukumar K. & Parihar N. S. Sequential Development of Histologic Lesions and Their Relationship with Bacterial Isolation, Fecal Shedding, and Immune Responses during Progressive Stages of Experimental Infection of Lambs with Mycobacterium avium subsp. paratuberculosis. Veterinary Pathology Online 41, 378–387 (2004). 10.1354/vp.41-4-378 15232138

[b28] Magombedze G., Eda S. & Koets A. Can Immune Response Mechanisms Explain the Fecal Shedding Patterns of Cattle Infected with *Mycobacterium avium* Subspecies *paratuberculosis*? PLoS ONE 11, e0146844 (2016). 26808389 10.1371/journal.pone.0146844 PMC4725749

[b29] Magombedze G., Eda S. & Ganusov V. V. Competition for Antigen between Th1 and Th2 Responses Determines the Timing of the Immune Response Switch during Mycobaterium avium Subspecies paratuberulosis Infection in Ruminants. PLoS computational biology 10, e1003414 (2014). 24415928 10.1371/journal.pcbi.1003414 PMC3886887

[b30] Magombedze G., Eda S. & Stabel J. Predicting the Role of IL-10 in the Regulation of the Adaptive Immune Responses in *Mycobacterium avium* Subsp. *paratuberculosis* Infections Using Mathematical Models. PLoS ONE 10, e0141539 (2015). 26619346 10.1371/journal.pone.0141539 PMC4664406

[b31] Stabel J. R. An Improved Method for Cultivation of Mycobacterium Paratuberculosis from Bovine Fecal Samples and Comparison to Three Other Methods. Journal of Veterinary Diagnostic Investigation 9, 375–380 (1997) 9376426 10.1177/104063879700900406

[b32] Stabel J. & Whitlock R. An evaluation of a modified interferon-γ assay for the detection of paratuberculosis in dairy herds. Veterinary immunology and immunopathology 79, 69–81 (2001). 11356251 10.1016/s0165-2427(01)00253-7

[b33] Wood P. . A field evaluation of serological and cellular diagnostic tests for bovine tuberculosis. Veterinary microbiology 31, 71–79 (1992). 1615636 10.1016/0378-1135(92)90142-g

[b34] Wood P. R. & Rothel J. S. *In vitro* immunodiagnostic assays for bovine tuberculosis. Veterinary microbiology 40, 125–135 (1994). 8073620 10.1016/0378-1135(94)90051-5

[b35] Doherty M., Bassett H., Quinn P., Davis W. & Monaghan M. Effects of dexamethasone on cell-mediated immune responses in cattle sensitized to Mycobacterium bovis. American journal of veterinary research 56, 1300–1306 (1995). 8928946

[b36] de Silva K. . Can early host responses to mycobacterial infection predict eventual disease outcomes? Preventive veterinary medicine 112, 203–212 (2013). 24034815 10.1016/j.prevetmed.2013.08.006

[b37] Stabel J. R. & Robbe-Austerman S. Early immune markers associated with Mycobacterium avium subsp. paratuberculosis infection in a neonatal calf model. Clin Vaccine Immunol 18, 393–405 (2011). 21228140 10.1128/CVI.00359-10 PMC3067369

[b38] Wigginton J. E. & Kirschner D. A model to predict cell-mediated immune regulatory mechanisms during human infection with Mycobacterium tuberculosis. J Immunol 166, 1951–1967 (2001). 11160244 10.4049/jimmunol.166.3.1951

[b39] Clark D. L. Jr., Koziczkowski J. J., Radcliff R. P., Carlson R. A. & Ellingson J. L. E. Detection of *Mycobacterium avium* Subspecies *paratuberculosis*: Comparing Fecal Culture Versus Serum Enzyme-Linked Immunosorbent Assay and Direct Fecal Polymerase Chain Reaction. Journal of dairy science 91, 2620–2627 (2008). 18565921 10.3168/jds.2007-0902

[b40] Stringer L. A. . Bayesian estimation of the sensitivity and specificity of individual fecal culture and Paralisa to detect Mycobacterium avium subspecies paratuberculosis infection in young farmed deer. Journal of Veterinary Diagnostic Investigation 25, 759–764 (2013). 24105379 10.1177/1040638713505587

[b41] Salgado M., Kruze J. & Collins M. T. Diagnosis of Paratuberculosis by Fecal Culture and ELISA on Milk and Serum Samples in Two Types of Chilean Dairy Goat Herds. Journal of Veterinary Diagnostic Investigation 19, 99–102 (2007). 17459841 10.1177/104063870701900117

[b42] Soetaert K. & Petzoldt T. Inverse modelling, sensitivity and monte carlo analysis in R using package FME. Journal of Statistical Software 33 (2010).

[b43] Taneja N. K., Dhingra S., Mittal A., Naresh M. & Tyagi J. S. *Mycobacterium tuberculosis* Transcriptional Adaptation, Growth Arrest and Dormancy Phenotype Development Is Triggered by Vitamin C. PLoS ONE 5, e10860 (2010). 20523728 10.1371/journal.pone.0010860 PMC2877710

[b44] Fratti R. A., Chua J., Vergne I. & Deretic V. Mycobacterium tuberculosis glycosylated phosphatidylinositol causes phagosome maturation arrest. Proceedings of the National Academy of Sciences 100, 5437–5442 (2003). 10.1073/pnas.0737613100 PMC154363 12702770

[b45] Clemens D. L. & Horwitz M. A. The Mycobacterium tuberculosis phagosome interacts with early endosomes and is accessible to exogenously administered transferrin. The Journal of Experimental Medicine 184, 1349–1355 (1996). 8879207 10.1084/jem.184.4.1349 PMC2192850

[b46] Schnappinger D. . Transcriptional adaptation of Mycobacterium tuberculosis within macrophages insights into the phagosomal environment. The Journal of experimental medicine 198, 693–704 (2003). 12953091 10.1084/jem.20030846 PMC2194186

[b47] Souza C., Davis W. C., Eckstein T. M., Sreevatsan S. & Weiss D. J. Mannosylated Lipoarabinomannans from *Mycobacterium Avium* Subsp. *Paratuberculosis* Alters the Inflammatory Response by Bovine Macrophages and Suppresses Killing of *Mycobacterium Avium* Subsp. *Avium* Organisms. PLoS ONE 8, e75924 (2013). 24098744 10.1371/journal.pone.0075924 PMC3786972

[b48] Kruh-Garcia N. A. . Detection of *Mycobacterium tuberculosis* Peptides in the Exosomes of Patients with Active and Latent *M. tuberculosis* Infection Using MRM-MS. PLoS ONE 9, e103811 (2014). 25080351 10.1371/journal.pone.0103811 PMC4117584

[b49] Schorey J. S. & Bhatnagar S. Exosome function: from tumor immunology to pathogen biology. Traffic 9, 871–881 (2008). 18331451 10.1111/j.1600-0854.2008.00734.x PMC3636814

[b50] Sweet L. . Mannose receptor-dependent delay in phagosome maturation by Mycobacterium avium glycopeptidolipids. Infection and immunity 78, 518–526 (2010). 19841083 10.1128/IAI.00257-09 PMC2798179

[b51] Mustafa T., Wiker H. G., Mørkve O. & Sviland L. Differential expression of mycobacterial antigen MPT64, apoptosis and inflammatory markers in multinucleated giant cells and epithelioid cells in granulomas caused by Mycobacterium tuberculosis. Virchows Archiv 452, 449–456 (2008). 18266005 10.1007/s00428-008-0575-z PMC2668550

[b52] Mustafa T., Wiker H., Mørkve O. & Sviland L. Reduced apoptosis and increased inflammatory cytokines in granulomas caused by tuberculous compared to non‐tuberculous mycobacteria: role of MPT64 antigen in apoptosis and immune response. Clinical & Experimental Immunology 150, 105–113 (2007). 17711491 10.1111/j.1365-2249.2007.03476.x PMC2219281

[b53] Reddy P. V., Puri R. V., Khera A. & Tyagi A. K. Iron storage proteins are essential for the survival and pathogenesis of Mycobacterium tuberculosis in THP-1 macrophages and the guinea pig model of infection. Journal of bacteriology 194, 567–575 (2012). 22101841 10.1128/JB.05553-11 PMC3264086

[b54] Kruh-Garcia N. A., Murray M., Prucha J. G. & Dobos K. M. Antigen 85 variation across lineages of Mycobacterium tuberculosis—Implications for vaccine and biomarker success. Journal of proteomics 97, 141–150 (2014). 23891556 10.1016/j.jprot.2013.07.005

[b55] Fernández M. . Macrophage Subsets Within Granulomatous Intestinal Lesions in Bovine Paratuberculosis. Veterinary Pathology, doi: 10.1177 (2016). 10.1177/0300985816653794 27315822

[b56] Dobson B., Liggett S., O’Brien R. & Griffin J. F. T. Innate immune markers that distinguish red deer (Cervus elaphus) selected for resistant or susceptible genotypes for Johne’s disease. Veterinary Research 44, 5–5 (2013). 23347398 10.1186/1297-9716-44-5 PMC3574005

