# Supplementary material for: Inferring biomarkers for Mycobacterium avium subsp. paratuberculosis infection and disease progression in cattle using experimental data
Source: Sci Rep. 2017 Mar 20;7:44765. doi: 10.1038/srep44765 (PMC5357903; doi:10.1038/srep44765)
Supplement: Supplementary Information [file srep44765-s1.pdf]

Inferring biomarkers for *Mycobacterium avium* subsp. *paratuberculosis* infection and disease progression in cattle using experimental data

Gesham Magombedze[1,2,4,*] **, Tinevimbo Shiri** [3] **, Shigetoshi Eda**[4,5] **, Judy R Stabel**[6]

[1] Center for Infectious Diseases Research and Experimental Therapeutics, Baylor Research Institute, Baylor University Medical Center, Dallas, TX, USA.

[2] Department of Infectious Disease Epidemiology. Imperial College London UK

[3] Warwick Clinical Trials Unit, Warwick Medical School, University of Warwick, Coventry, UK

[4] National Institute for Mathematical and Biological Synthesis,

[5] Department of Forestry, Wildlife, and Fisheries, University of Tennessee, Knoxville, TN 37996-1527 USA

[6] USDA-ARS, National Animal Disease, Ames, IA 50010 USA

Supplementary Text: S1

Supplementary methods

Model fitting

A Bayesian frame work was used to fit the models to data in R using the FME package [1] based on the procedure by [2] that implements the delayed rejection and adaptive metropolis algorithm simulated using the Markov Chain Monte Carlo (MCMC) method. Prior estimates of the model parameters were taken from our previous studies [3-5]. For each parameter, a uniform prior was used with the lower and upper values taken as the parameter bounds and the median as the parameter estimate. In the fitting we assumed that the observations have identically and independently distributed additive Gaussian noise with unknown variance. Therefore, given data/observations D, and a nonlinear model $M, \left(M = f(x,\theta)\right)$, the data can be explained as $D = f(x,\theta) + error$, where $error = N(0,\delta^2)$ is the Gaussian noise. θ are the model parameters to be estimated and x are the model time dependent variables. Applying the Bayesian probability, the model parameters can be drawn from the posterior distribution $P(D/M\,,\delta^2) = \frac{1}{P(D)} * P(M/D)P(M)$, where $P(M)$ is the model prior, $P(D)$ is the model evidence which in practice amounts to a normalising constant and $P(M/D)$ is the model likelihood. Assuming uniform priors we estimated model parameters from posterior distributions generated using the MCMC method and a Gaussian likelihood function, $L(M,D)$, therefore

$$L(M,D) \propto \prod_{i=1}^{n} \exp(-(\boldsymbol{D_i} - \boldsymbol{M_i})^2/2\boldsymbol{\delta}^2)$$

$$= \prod_{i=1}^{n}\left[exp\left(\frac{-(Th1_i - IFN_i)^2}{2\delta_1^2}\right)exp\left(\frac{-(Th2_i - ELISA_i)^2}{2\delta_2^2}\right)exp\left(\frac{-(CFU_i - CFU_{datai})^2}{2\delta_3^2}\right)\right]$$

This likelihood function was used in estimating parameters for the model in the two different modelling approaches, where $Th1_i, Th2_i, CFU_i$, represent the model simulated Th1, Th2 and CFU values and $IFN_i, ELISA_i, CFU_{datai}$,are the observations from the study and δ_is are the variances.

Selecting parameters for model fitting

The parameters k_i, k_b, $\theta_1, \theta_2, \theta_3$, λ_1 and μ_f were selected for model fitting based on our previous studies were we carried out model parameter sensitivity analysis [3-5]. In the Hybrid model, the CFU shedding parameters in the ODE model were replaced with the parameters π_0, π_1 and π_2 in the logistic shedding function. Also, in both model we estimated the initial values for macrophages ($M_\phi(0)$, $I_m(0)$, $B(0)$, since the actual time/day of infection is not known.

Chain convergence diagnostics

We used the Coda R package [6] to evaluate the convergence of the MCMC chain. First, we visually assessed the chains to determine if stationarity was achieved. We then used the Geweke's diagnostic, which compares the 10% first part of the chain with the 50% last part of the chain, these should be the same. The test calculates a z-score for the difference in the means between the two parts of the chains. Our chains returned values between 2 and -2, which indicates the two means of the chain are not that different.

Calculating of assay sensitivity cut-off points

Assay sensitivity cut-off values in the simulations were calculated using experimental assay sensitivity cut-off values (see Table S2). The macrophage cut-off value was set at the value of the IFN-γ assay cut off since a Th1/IFN-γ response is stimulated by infected macrophages or by intracellular infection. We used the maximum observed values to normalise the data to a scale of 0 to 1 for the measured values (Table S2). The models were fitted to the normalised data and the normalised cut-off values were used as the sensitivity cut-off in the simulation of the assays.

Supplementary Figures and Tables

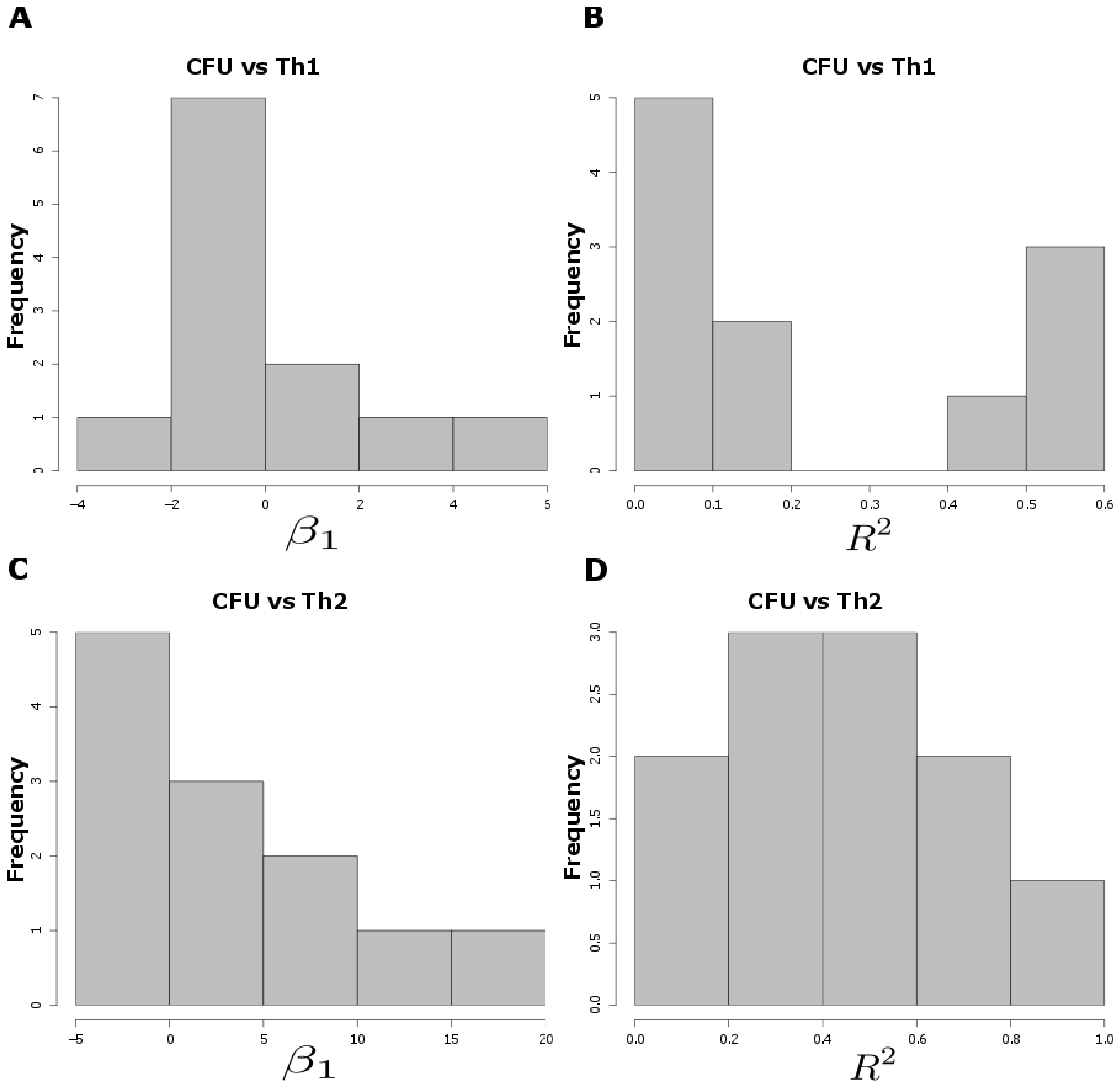

Fig S1: Relationships between IFN- γ (Th1), ELISA (Th2) and CFUs. Histograms illustrating the distribution of linear regression, $y = \beta_0 + \beta_1 x$, coefficients (slope of the fitted line, β_1) and the coefficient of determination (R^2) of the level of correlation between Th1 vs CFUs, and Th2 vs CFUs. **A** and **C**, show the distribution of the regression coefficient, β_1. **B** and **D**, the coefficient of determination of the level of correlation between the Th1 and Th2 responses with the CFU shedding.

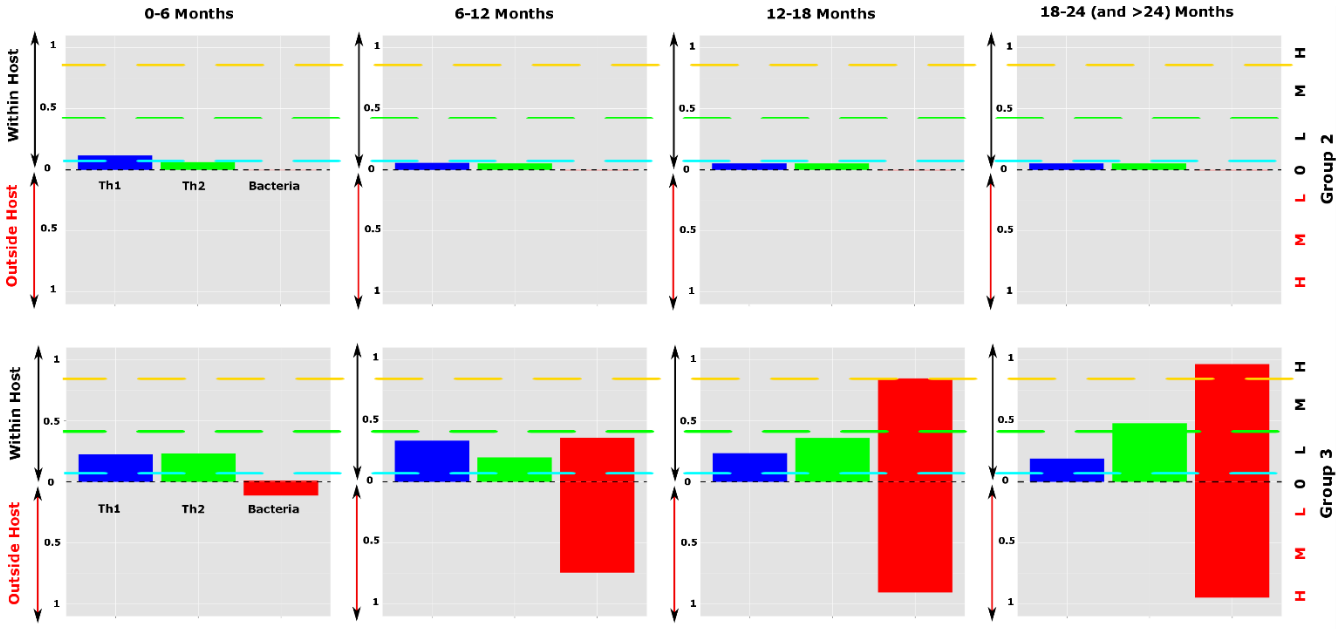

Fig S2: Theoretical assay predictions. Model predictions showing how Th1, Th2 and CFU based assays will probably show when assay sample are taken at different time intervals, for instance using 6 month sliding windows. This simulation also shows the level of within host free bacteria, which cannot be easily tested. However, here, we present a prediction of the probable within host free bacteria that corresponds to the excreted bacteria that is normally detected using the faecal culture assay. Group 2 animals show that, no CFU will be excreted and no free bacteria can be isolated from within the animals. However, lowly expressed Th1 and Th2 responses, suggest the presence of an infection.

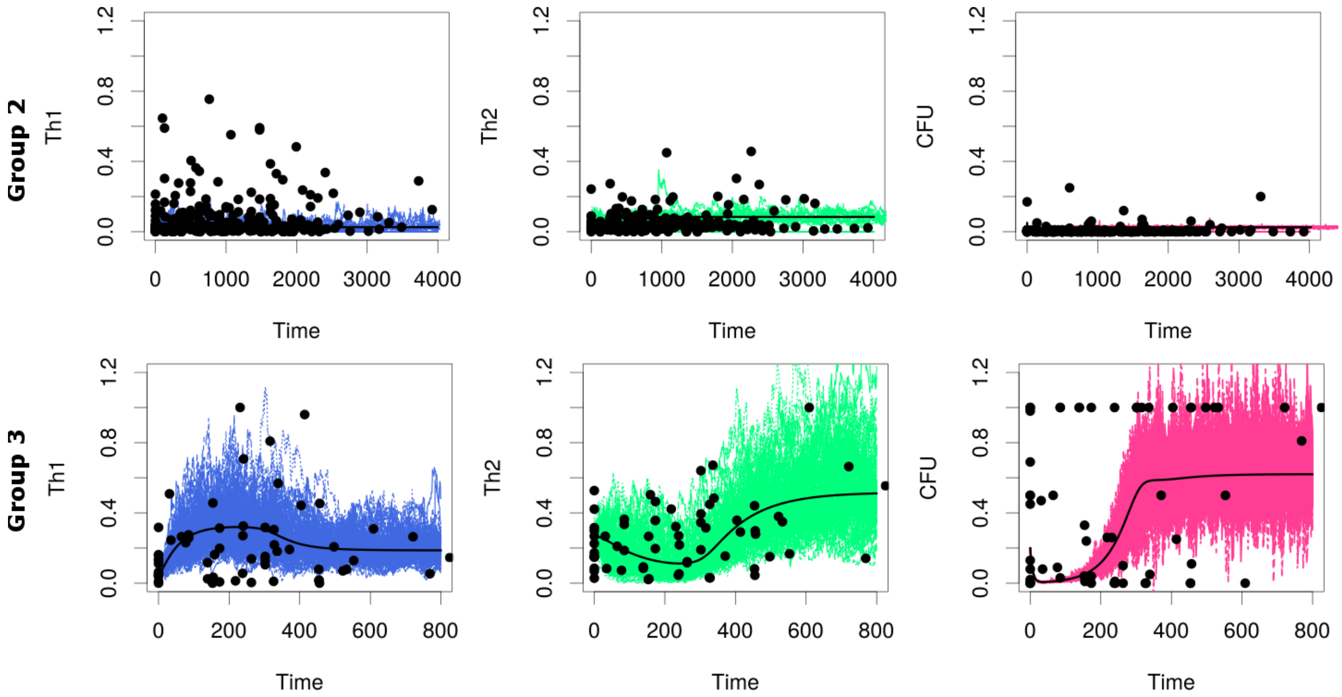

Fig S3: Data fitting with a stochastic model. An illustration of how the stochastic model explains the IFN- γ (Th1) and ELISA (Th2) and CFUs time evolution for animals in groups 2 and 3, respectively. The estimated parameters from the ODE fitting were used with different levels of stochastic signal between the two groups. A total of 100 runs were simulated to illustrate the spectrum and impact of stochastic immune response stimulation and CFU shedding.

Table S1: **Model parameters**. We list parameters that have been derived using information from the following literature sources.

Name	Definition	Dimension	Range/prior	Baseline Value
δ_m	Macrophage supply	cell/mm^3/day	5.0-10.0	10.0
μ_m	Macrophages death rate	day^{-1}	0.11-0.025	0.02
μ_I	Infected macrophages death rate	day^{-1}	0.11-0.025	0.02
μ_B	Bacteria death rate	day^{-1}	0.0-1.0	0.03
k_i	Macrophage infection rate	mm^3/cell/day	0.0-1.0	0.002
k_m	Bacteria removal by macrophages	mm^3/cell/day	0.0-1.0	0.000125
k_b	Infected macrophages burst rate	day^{-1}	0.0-1.0	0.00075
N_0	Burst size	-	80.0-100.0	100.0
μ_0	Th0 decay/death rate	day^{-1}	0.1-0.03	0.01
μ_1	Th1 decay/death rate	day^{-1}	0.1-0.03	0.03
μ_2	Th2 decay/death rate	day^{-1}	0.01-0.02	0.02
δ_0	Th0 supply	cell/mm^3/day	0.1-1.0	0.1
θ_1	Th1 cells clonal expansion	-	1.0-9000.0	1000.0
θ_2	Th2 cells clonal expansion	-	1.0-9000.0	1000.0
δ_m	Th0 differentiation into Th1 cells	mm^3/cell/day	0.0-1.0	0.01
δ_B	Th0 differentiation into Th2 cells	mm^3/cell/day	0.0-1.0	0.01
k_l	Th1 lytic effect	mm^3/cell/day	0.0-0.2	0.00004
λ_1	Bacteria shedding	day^{-1}	0.0-1.0	0.002
λ_2	Bacteria shedding	day^{-1}	0.0-1.0	0.002
μ_f	CFU decay rate	day^{-1}	0.01-0.02	0.02

Table S2: Calculation of assay sensitivity cut-off values

Assay	Sensitivity experimental cut-off value (SEV)	Maximum measured value (MMV)	Normalised cut-off value (NV=SEV/MMV)
IFN-γ	>0.1	2.348	~0.04
ELISA	>0.25	2.561	~0.1
CFU	>1	100	0.01
Macrophages	-	-	0.04 (Set to IFN-γ value)

Table S3: Predicted correlations. Estimated level of correlations predicted using linear models/linear regression.

	Group1			Group 2			Group 3		
	R^2 Adj	p-value	F-test	R^2 Adj	p-value	F-test	R^2Adj	p-value	F-test
$y = \beta_1 x$									
CFUs-vs-ELISA	0.022	1.8e-2	5.72	0.05	<0.001	14.69	0.35	<0.001	171.91
CFUs-vs-IFN-γ	0.043	1.6e-3	10.23	0.019	0.015	6.0	0.14	<0.001	52.59
ELISA-vs-IFN-γ	0.14	1.8e-8	34.27	0.13	<0.001	40.68	0.28	<0.001	122.08
$y = \beta_0 + \beta_1 x$									
CFUs-vs-ELISA	-4.3e-3	0.74	0.11	6.0e-3	0.12	2.48	0.26	<0.001	112.89
CFUs-vs-IFN-γ	1.4e-3	0.26	1.28	-3.8e-3	0.83	0.05	0.05	<0.001	18.55
ELISA-vs-IFN-γ	-4.8e-3	0.9	1.6e-2	-1.2e-3	0.40	0.70	0.10	<0.001	33.58

References

1 Soetaert, K. & Petzoldt, T. Inverse modelling, sensitivity and monte carlo analysis in R using package FME. *Journal of Statistical Software* **33** (2010).

2 Haario, H., Laine, M., Mira, A. & Saksman, E. DRAM: Efficient adaptive MCMC. *Statistics and Computing* **16**, 339-354 (2006).

3 Magombedze, G., Eda, S. & Koets, A. Can Immune Response Mechanisms Explain the Fecal Shedding Patterns of Cattle Infected with *Mycobacterium avium* Subspecies *paratuberculosis*? *PLoS ONE* **11**, e0146844 (2016).

4 Magombedze, G., Eda, S. & Ganusov, V. V. Competition for Antigen between Th1 and Th2 Responses Determines the Timing of the Immune Response Switch during Mycobaterium avium Subspecies paratuberulosis Infection in Ruminants. *PLoS computational biology* **10**, e1003414 (2014).

5 Magombedze, G., Eda, S. & Stabel, J. Predicting the Role of IL-10 in the Regulation of the Adaptive Immune Responses in *Mycobacterium avium* Subsp. *paratuberculosis* Infections Using Mathematical Models. *PLoS ONE* **10**, e0141539 (2015).

6 Plummer, M. & Best, N. Package 'coda'. (2015).
